# Slow Titration of Cannabidiol Add-On in Drug-Resistant Epilepsies Can Improve Safety With Maintained Efficacy in an Open-Label Study

**DOI:** 10.3389/fneur.2020.00829

**Published:** 2020-08-12

**Authors:** Gianluca D'Onofrio, Mathieu Kuchenbuch, Caroline Hachon-Le Camus, Béatrice Desnous, Véronique Staath, Sylvia Napuri, Dorothée Ville, Jean-Michel Pedespan, Anne Lépine, Claude Cances, Anne de Saint-Martin, Théo Teng, Nicole Chemaly, Mathieu Milh, Nathalie Villeneuve, Rima Nabbout

**Affiliations:** ^1^Department of Pediatric Neurology, Reference Centre for Rare Epilepsies, Hôpital Necker-Enfants Malades, APHP, Paris, France; ^2^Pediatric Residency, Department of Women and Child Health, University of Padua, Padua, Italy; ^3^Laboratory of Translational Research for Neurological Disorders, INSERM UMR 1163, Imagine Institute, Université de Paris, Paris, France; ^4^Department of Pediatric Neurology, Hôpital des Enfants, Toulouse University Hospital, Toulouse, France; ^5^AP-HM, Pediatric Neurology Department, Timone Children Hospital, Marseille, France; ^6^Department of Pediatric Neurology, Strasbourg University Hospital, Hopital de Hautepierre, Strasbourg, France; ^7^Department of Pediatrics, Rennes University Hospital, University Rennes, Rennes, France; ^8^Department of Pediatric Neurology, CNRS UMR 5304, Bron, France; ^9^Department of Pediatrics, Pellegrin University Hospital, Bordeaux, France

**Keywords:** Dravet, Lennox-Gastaut, adverse events, liver function, tolerability, drug resistant

## Abstract

**Objective:** To assess adverse events (AEs) and efficacy of add-on cannabidiol (CBD) with a slower titration protocol in pediatric clinical practice.

**Methods:** We conducted a prospective, open-label, multicenter study in seven French reference centers for rare epilepsies. Patients had slow titration to reach a target dose of 10 mg/kg/day within at least 1 month and then gradually increased to a maximum dose of 20 mg/kg/day. We analyzed AEs and efficacy at M1 (month 1), M2, and M6, comparing two sets of subgroups: Dravet syndrome (DS) vs. Lennox-Gastaut (LGS) and patients with clobazam (CLB+) vs. patients without (CLB−).

**Results:** One hundred and twenty-five patients were enrolled (62 LGS, 48 DS, 5 Tuberous sclerosis, and 10 other etiologies). Median concomitant antiepileptic drugs (AEDs) was three (25th percentile: 3, 75th percentile: 4). Patients received a dose of 10 (10–12), 14 (10–20), and 15.5 mg/kg/day (10–20) at M1, M2, and M6, respectively. Twenty-six patients discontinued CBD, 19 due to lack of efficacy, 2 due to AEs, 4 for both, and 1 had a sudden unexpected death in epilepsy. AEs were reported in 61 patients (48.8%), mainly somnolence (*n* = 26), asthenia (*n* = 20), and behavior disorders (*n* = 16). Abnormal transaminases (≥3 times) were reported in 11 patients receiving both valproate and clobazam. AEs were significantly higher at M2 (*p* = 0.03) and increased with the number of AEDs (*p* = 0.03). At M6, total seizure frequency change from baseline was −41% ± 37.5% (mean ± standard deviation), and 28 patients (37.8%) had a reduction ≥50%. AE and efficacy did not differ between DS vs. LGS and CLB+ vs. CLB– patients.

**Significance:** A slower titration of CBD dose delivered better tolerance with comparable efficacy to previous trials. Concomitant CLB did not increase efficacy rates but in a few cases increased AEs. This slow titration scheme should help guide clinicians prescribing CBD and allow patients to benefit from its potential efficacy.

## Introduction

Since June 2018, an oil-based, highly purified, liquid formulation of cannabidiol (CBD, Epidiolex® in the United States, Epidyolex® in the European Union) was approved by the Food and Drug Administration (FDA) for the treatment of seizures associated with DS and LGS in individuals 2 years of age and older (1). In France, CBD had been available since December 2018 in the form of a nominative “temporary authorization for use” (ATU) (2) and more recently (September 2019) via marketing authorization of Epidyolex® by the European Medicines Agency (EMA) (3). The indication was given for “adjunctive therapy of seizures associated with LGS and DS, in conjunction with clobazam (CLB), for patients 2 years of age and older” (3). Within the nominative ATU (ATUn) use, prescription of CBD has been extended to selected drug-resistant patients.

CBD is a non-psychoactive compound of cannabis with promising anticonvulsant properties. The antiepileptic mechanisms of CBD are not known, but it would seem to interact with many signaling systems: antagonism of G protein-coupled receptor 55 (GPR55), desensitization of transient receptor potential of vanilloid type 1 (TRPV1) channels, and inhibition of adenosine reuptake (4). Moreover, CBD has neuroprotective and anti-inflammatory effects (5).

Four randomized controlled trials (RCT) were designed to assess CBD safety and efficacy in DS (GWPCARE 1 and 2) (6–8) and LGS (GWPCARE 3 and 4) (9, 10). Patients with DS and LGS who had previously participated in RCTs were enrolled in an open-label extension trial (OLE) (GWPCARE 5) (11, 12). A fifth RCT of CBD (GWPCARE 6) for the treatment of seizures associated with tuberous sclerosis complex (TSC) is ongoing with preliminary positive results (13). Data on CBD use in other drug-resistant epilepsies come from open-label studies (OL) (14, 15) and expanded-access programs (EAP) (16), which include patients with Aicardi syndrome, CDKL5 mutation, Doose syndrome, dup15q disorders, Febrile infection–related epilepsy syndrome, and other epilepsy syndromes. A median percentage reduction in total seizures has been estimated between 28.6 and 57% (6, 10) (Supplementary Table 3). The safety profile reported in all the studies is rather homogeneous with a percentage of AEs between 74 and 93% (6, 7) (Supplementary Table 2). The most common AEs were somnolence, decreased appetite, increase in transaminases, diarrhea, and fatigue (6, 7, 9–11, 14, 16). These AEs sometimes led to treatment discontinuation, in particular due to transaminases increase, which was correlated with concomitant valproate (VPA) use (17). These studies were designed to reach a target dose of CBD of 20–25 mg/kg/day until 50 mg/kg/day (13, 14, 16) in 2–4 weeks.

Here, we hypothesize that the use of a slower titration protocol of CBD might improve CBD tolerance without affecting its efficacy. Thus, we conducted a real-life study to report the efficacy and tolerance of the introduction of CBD, using a slower titration protocol associated with the possibility of modifying the comedications, according to the clinical, biological, and tolerance data reported by families and practitioners. We also aimed to compare the outcome in patients with DS and LGS and in patients with and without CLB (CLB+ vs. CLB−).

## Methods

### Participants

We conducted a prospective, open-label, multicenter study involving seven national centers in the network of French Reference Centers for Rare Epilepsies (Bordeaux, Lyon, Marseille, Paris Necker Enfants Malades, Rennes, Strasbourg, Toulouse). We included patients aged 2–18 years with drug-resistant epilepsy, receiving at least one antiepileptic drug with no therapeutic changes (including for vagus nerve stimulator or ketogenic diet) for ≥4 weeks, starting pharmaceutical formulation of purified CBD (100 mg/mL) in oral solution (Epidyolex; GW Research Ltd) between March and September 2019.

This study was approved by the ethics committee of our institution Necker Hospital, APHP. All participants or their legal guardians signed an informed consent to participate in this study.

### Study Design

CBD prescription was conditional upon obtaining the formal agreement through ATUn. Patients had a 4 week baseline period during which caregivers completed seizure diaries. CBD was administered orally twice daily in equally divided doses, starting at 2.5 mg/kg/day and increasing by 2.5 mg/kg/day weekly with a target dose of 10 mg/kg/day after 4 weeks. Dose was then gradually increased between the first and the sixth month, according to tolerance and efficacy, to a maximum of 20 mg/kg/day.

Safety and efficacy of CBD was evaluated during outpatient clinics in the different sites at 1 (M1), 2 (M2), and 6 months (M6). We reported and analyzed AEs for all patients. The safety of CBD was estimated, using detailed checklists, with clinical examination, liver tests, and monitoring incidences and types of AEs. For CBD efficacy, we reported all patients and analyzed patients with more than four seizures per month. Indeed, below this threshold, evaluation of CBD efficacy over a period of 6 months was not considered relevant. The efficacy of CBD was determined by (1) change in total monthly seizure frequency, (2) seizure reduction responder rates (proportion of patients who had at least 50, 70, and 90% reduction in total monthly seizures), and (3) caregivers and (4) physician Clinical Global Impression-Improvement scale (CGI-I) scale scores. The CGI-I scale uses a Likert method with responses in seven categories from 1 for “very much worse” to 7 for “very much improved,” and 4 for “no change from baseline.”

Total monthly seizure frequency was recorded by caregivers and practitioners and expressed as a percentage of baseline. Finally, we also collected the antiepileptic drug (AED) modifications carried out at each visit.

### Statistical Analysis

We used descriptive analysis to characterize our population. Assessed variables included gender, age at onset and at CBD introduction, number of concomitant AEDs, most frequent concomitant AEDs, epileptic syndrome, seizure type (generalized, focal, both), and baseline monthly seizure frequency. Normal and non-normal data were represented as mean ± standard deviation (SD) and median (25th−75th percentiles), respectively. To compare DS vs. LGS and CLB+ vs. CLB−, we used a Wilcoxon non-parametric test for numerical variables and performed contingency analysis with chi-squared test or Fisher's exact test (2 × 2 levels) for categorical variables with a Bonferroni correction if necessary.

Changes in the dosage (mg/kg/day) between M1, M2, and M6 were studied using mixed-effects ANOVA for repeated measures due to variations in participant number. Patient ID was fitted as a random effect, and time at CBD evaluation was fitted as a fixed factor. CLB status and epileptic syndrome factors (DS/LGS) were added into this statistic model to study the potential impact of CLB and epileptic syndrome, respectively. A *post-hoc* Tukey's test followed ANOVA in the case of statistical significance.

To assess the efficacy of CBD, we used mixed ANOVA for repeated measures as described above to study the evolution of monthly seizure frequency and CGI-I scores in the general population and according to CLB status and epilepsy syndrome. Then, we analyzed factors associated with a decreased seizure frequency below 50% of the baseline during the treatment period through a mixed logistic regression. Factors included in this analysis were age at CBD introduction, epilepsy duration, epileptic syndrome (DS, LGS), gender, dose of CBD, time (M1, M2, and M6), and the five most frequently associated AEDs [VPA, CLB, stiripentol (STP), topiramate (TPM), lamotrigine (LTG)]. First, we performed univariate analysis, and then we included the significant factors in a multivariate analysis.

The safety of the CBD has been assessed through the presence of AE. We first described their rates and types during the different evaluation time points throughout our cohort. Then we compared DS vs. LGS and CLB+ vs. CLB− groups with the statistical methods described above. We used mixed logistic regressions to identify factors statistically associated with AE and efficacy. The factors included were the same as those in the logistic regression conducted to assess effectiveness.

Finally, through logistic regressions, we studied the relation between the presence of AEs and parents' CGI-I score and between the presence of AEs and change in estimated seizure rates (%) at M6.

## Results

### Population

Between March 2019 and September 2019, 125 patients were enrolled. All patients had received CBD through ATUn. We had 90.2% of the requested data for this cohort (missing 408 of 4,161 data entries).

The patients treated had LGS (*n* = 62, 49.6%), DS (*n* = 48, 38.4%), TSC (*n* = 5, 4%), and other epilepsy syndromes (*n* = 10, 8%), including three SYNGAP1-epilepsy and three with epilepsy with migrating focal seizures in infancy (Table 1). At baseline, 28 patients (22.4%) had several seizure types that were difficult for caregivers to quantify. In the other 97 patients (77.6%), monthly seizure frequency was 30.5 (25th−75th percentile: 5–122). Most patients had generalized seizures (*n* = 61, 48.8%) or both focal and generalized (*n* = 45, 36%), and 14 patients (11.2%) had only focal seizures. Drop-seizures were reported in 62 patients (49.6%). Age at onset of epilepsy was 8 (4–34.5) months, and age of CBD initiation was 9 (6–14) years. Patients were receiving a median of three AEDs (3–4) concomitantly; the most common were VPA (*n* = 81, 64.8%), CLB (*n* = 77, 61.6%), TPM (*n* = 42, 33.6%), STP (*n* = 38, 30.4%), and LTG (*n* = 32, 25.6%).

**Table 1:** Basic demographic and clinical characteristics of patients (on the left) and comparisons of subpopulations of patients with Dravet and Lennox-Gastaut syndromes (in the middle) and with/without Clobazam (on the right).

**Variable**	**Total *N* = 125**	**DS *N* = 48 38.4%**	**LGS *N* = 62 49.6%**	**DS vs. LGS *p***	**CLB+ *N =* 77 61.6%**	**CLB− N = 48 38.4%**	**CLB+ vs. CLB−*p***
Onset age of epilepsy (months)	8 [4–34.5]	6 [4–7.8]	24 [8.5–60]	<10^−4^	5 [8–30]	4 [9–36]	ns
Age of CBD introduction (years)	9 [6–14]	7 [5–10.3]	13 [8.25–15.8]	<10^−4^	5 [9–14]	10 [6–14]	ns
Male sex	59 (47.2%)	18 (37.5%)	35 (56.4%)	0.07	41 (53.9%)	17 (35.4%)	0.01
**Seizures types repartition**							
Focal	14 (11.2%)	5 (10.4%)	4 (6.5%)	ns	7 (9.1%)	7 (14.6%)	ns
Generalized	61 (48.8%)	24 (50%)	36 (58.1%)	ns	40 (51.9%)	21 (43.7%)	ns
Both	45 (36.0%)	16 (33.3%)	20 (32.3%)	ns	26 (33.8%)	19 (39.6%)	ns
Falling seizure	62 (49.6%)	16 (33.3 %)	41 (66.1%)	0.01	38 (49.3%)	24 (50%)	ns
Number of concomitant AEDs	3 [3–4]	3 [3–4]	3 [3–4]	ns	3 [3–3]	3 [2.25–4]	0.002
**Main concomitant AEDs**							
VPA	81 (64.8%)	39 (81.3%)	40 (64.5%)	0.09	56 (72.7%)	25 (52.1%)	0.07
CLB	76 (60.8%)	37 (77.1%)	33 (53.2%)	0.04	77 (100%)	0 (0%)	<10^−4^
TPM	42 (33.6%)	24 (50%)	16 (25.8%)	0.007	27 (35.1%)	15 (31.3%)	ns
STP	38 (30.4%)	38 (79.2%)	0 (0%)	<10^−4^	32 (41.6%)	6 (12.5%)	0.001
LTG	38 (30.4%)	0 (0%)	34 (54.8%)	<10^−4^	22 (28.6%)	16 (33.3%)	ns
**Category of epileptic syndrome**							
LGS	62 (49.6%)	–	62 (100%)	<10^−4^	33 (42.9%)	29 (60.4%)	ns
DS	48 (38.4%)	48 (100%)	–	<10^−4^	38 (49.3%)	10 (20.8%)	<0.05
TSC	5 (4%)	–	–		3 (3.9%)	2 (4.2%)	ns
Other	10 (8%)	–	–		3 (3.9%)	7 (14.6%)	<0.05
Baseline seizure frequency (months)	30.5 [5–122]	5.5 [3.9–13.5]	91.5 [38.3–236.4]	<10^−4^	9 [4–153]	61 [17–145]	0.03
Several per days seizures	28 (22.4%)	2 (4.2%)	25 (40.3%)	<10^−4^	17 (22.1%)	11 (22.9%)	ns

*CBD, Cannabidiol; DS, Dravet Syndrome; LGS, Lennox-Gastaut Syndrome; TSC, Tuberous sclerosis complex; AEDs, anti-epileptic drugs; CLB, clobazam; VPA, valproate; STP, stiripentol; TPM, topiramate; LTG, lamotrigine*.

**Figure 1:**
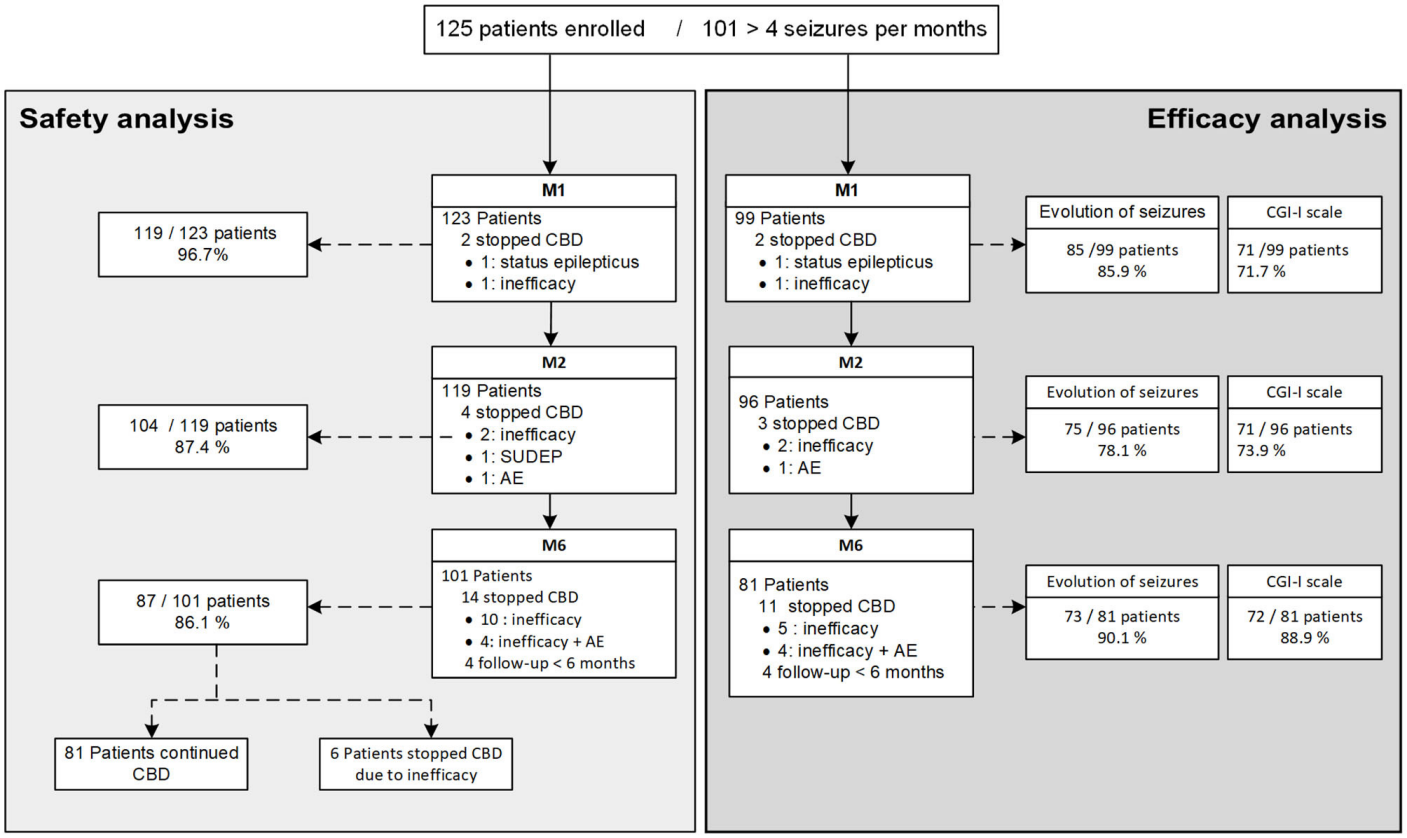
Flow chart of the study, including safety and efficacy. CBD, Cannabidiol; CGI-scale, Clinical Global Impression-Improvement scale; AE, Adverse Events; SUDEP, Sudden Unexpected Death in Epilepsy; M1, 1 month of treatment; M2, 2 months of treatment; M6, 6 months of treatment.

At M1, 98.4% remained on CBD, 95.2% at M2, and 80.8% at M6 (flowchart, Figure 1). Safety analyses were performed on 96.7% of M1 patients (*n* = 119), 87.4% of M2 patients (*n* = 104), and 86.1% of M6 patients (*n* = 87). Efficacy was assessed on 71.7% (*n* = 71), 73.9% (*n* = 71), and 88.9% (*n* = 72) for CGI-I and on 85.9% (*n* = 85), 78.1% (*n* = 75), and 90.1% (*n* = 73) for change in seizure frequency at M1, M2, and M6, respectively.

**Figure 2:**
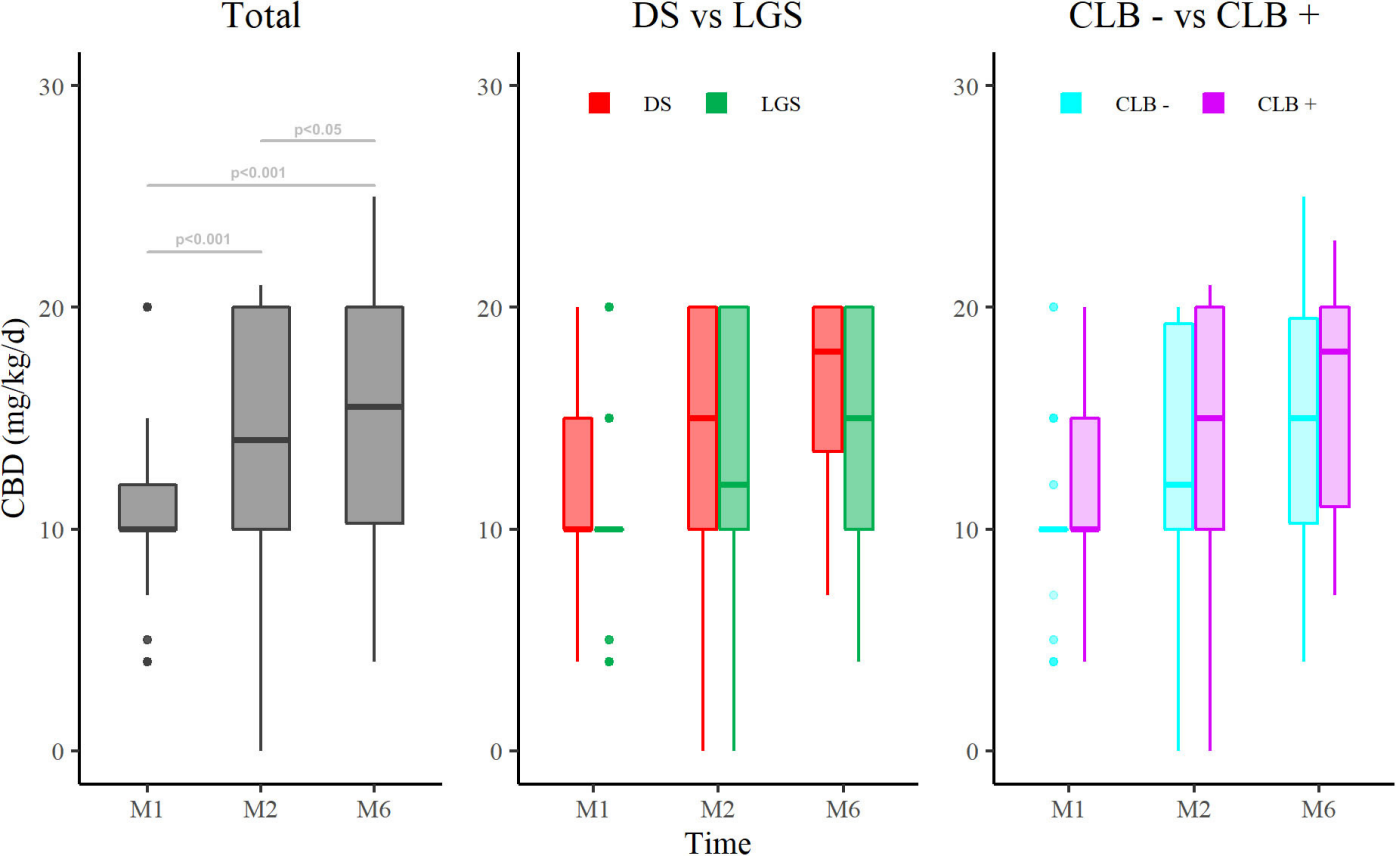
Evolution of cannabidiol dose at M1, M2, and M6 after introduction of CBD in the total population (**left** panel), in DS and LGS (**middle** panel), and in patients with CLB+ and CLB− comedication (**right** panel). CBD, cannabidiol; M1, 1 month of treatment; M2, 2 months of treatment; M6, 6 months of treatment; DS, Dravet Syndrome; LGS, Lennox-Gastaut syndrome; CLB− patients without clobazam, CLB+ patients with clobazam. *P < 0.05, **P < 0.01, ***P < 0.001.

The median CBD dose increased significantly between M1 [10 (10–12) mg/kg/day], M2 [14 (10–20) mg/kg/day], and M6 [15.5 (10–20) mg/kg/day] (Figure 2). The DS subpopulation received a higher dose compared to LGS (*p* < 0.05). No difference was observed between CLB+ and CLB− subgroups (Figure 2).

**Figure 3:**
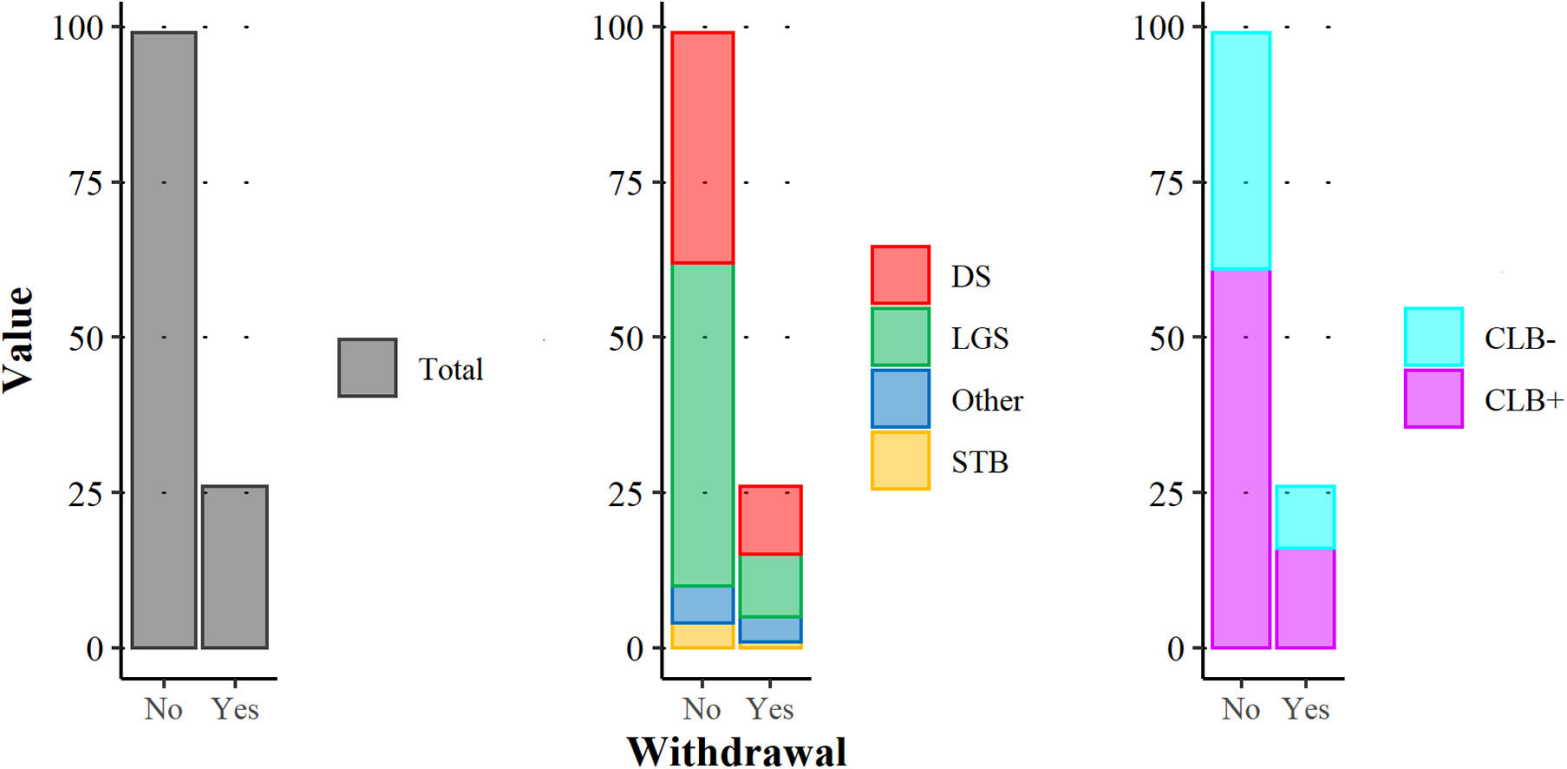
CBD withdrawal during this study in the total population (**left** panel), according to the category of epileptic syndrome (**middle** panel) and to CLB status, CLB+ vs. CLB− (**right** panel). DS, Dravet Syndrome; LGS, Lennox-Gastaut syndrome; CLB− patients without clobazam, CLB+ patients with clobazam.

Twenty-six patients (20.8%) stopped the treatment following a median of 3 months (25th−75th percentile: 1.8–3.8), mostly due to lack of efficacy (*n* = 19/26, 73.1%), four patients (15.4%, *n* = 4/26) for both lack of efficacy and AEs. Two patients (7.7%, *n* = 2/26) stopped CBD for AEs; one had convulsive status epilepticus hours after the first dose of CBD and the other for behavioral changes after 1.5 months of treatment. There was one sudden unexpected death in epilepsy a month after CBD introduction in a 2 year-old patient with DS. The difference in withdrawal of DS vs. LGS and CLB− vs. CLB+ was not significant (Figure 3).

### Safety

**Figure 4:**
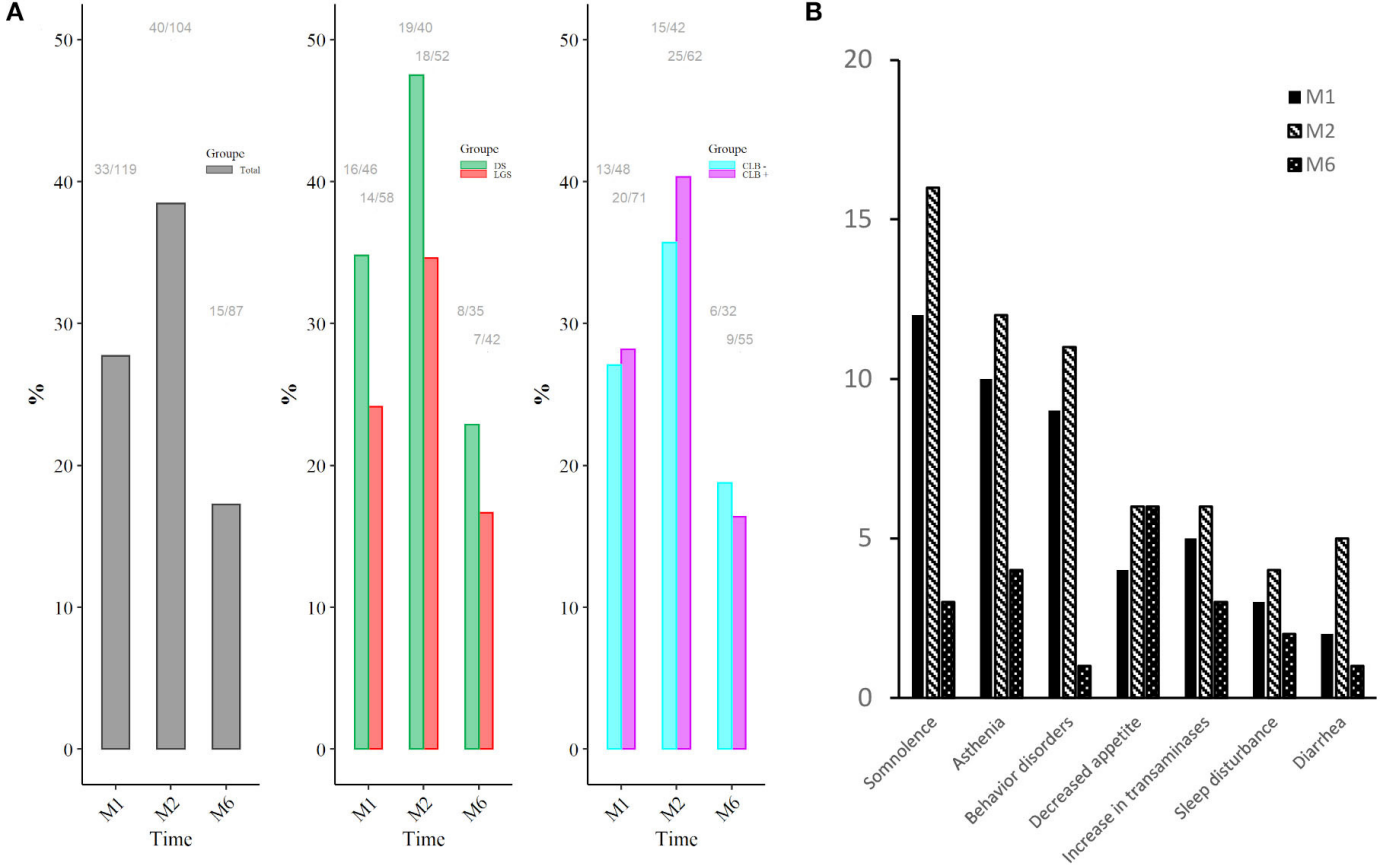
Adverse event rates as a function of time **(A)** in the total population (left side), comparing DS and LGS (middle side), and patients CLB+ and CLB− (right side) and types and incidence of adverse events reported **(B)**. M1, 1 month of treatment; M2, 2 months of treatment; M6, 6 months of treatment; DS, Dravet Syndrome; LGS, Lennox-Gastaut syndrome; CLB− patients without clobazam, CLB+ patients with clobazam. The same patient could have the same AE during the whole treatment period.

AEs were reported in 61 patients (48.8%) during the whole period of follow-up. Thirty-three (27.7%) patients presented AEs at M1, 40 at M2 (38.5%), and 15 at M6 (17.2%) (Figure 4A). No significant difference in incidence, type, or distribution of AE was observed in DS vs. LGS and CLB+ vs. CLB−. The most relevant AEs were somnolence (*n* = 26, 20.8%), asthenia (*n* = 20, 16%), behavior disorders (*n* = 16, 12.8%), decreased appetite (*n* = 12, 9.6%), sleep disturbance (*n* = 7, 5.6%), and diarrhea (*n* = 6, 4.8%). The highest level of AEs was at M2 (Figure 4B).

Increases in transaminases, aspartate aminotransferase (AST) or alanine aminotransferase (ALT), defined as greater than three times the upper limit of normal (ULN), was observed in 11 patients (9.6%), up to a maximum of seven times the upper limit. All patients with increased transaminases were receiving both VPA and CLB. Normalization of hepatic enzymes was observed in six patients. They had a decrease in antiepileptic drugs (VPA in two, VPA+CLB in two, VPA+STP in one, and CBD in one). None discontinued treatment due to hepatotoxicity, and no patient met the Hy's law criteria for serious drug-induced liver injury (18).

Three different strategies have been used by practitioners to face the emergence of these AEs at M1 and M2: (1) no change in treatment (54.5%, i.e., *n* = 18/33, at M1 and 47.5%, i.e., *n* = 19/40, at M2), (2) a decrease in comedication (39.4%, i.e., *n* = 13/33 and 42.5%, i.e., *n* = 18/40, at M1 and M2, respectively), or (3) a decrease in CBD (0.9%, i.e., *n* = 3/33 and 20%, i.e., *n* = 8/40 at M1 and M2, respectively); sometimes the last two were combined (one case at M1 and five at M2). AEs resolved spontaneously in 33.3% at M2 (*n* = 6/18) and 52.6% at M6 (*n* = 10/19). AEs resolved with comedication decrease in 46.2% at M2 (*n* = 6/13) and 52.9% at M6 (*n* = 9/17). CBD decrease in three patients with AEs at M2, including a decrease in comedication in one, did not result in AE resolution. On the contrary, CBD decrease helped to resolve AEs in 6/8 patients at M6, including in five a concomitant decrease of comedications.

In multivariate regression analysis, AEs were significantly higher in M2 (OR adj. 2.23, CI_95%_: 1.06–4.67, *p* = 0.03), and a higher number of AEDs was significantly associated with AEs (OR adj. 1.74, CI_95%_: 1.06–2.85, *p* = 0.03).

### Efficacy

**Figure 5:**
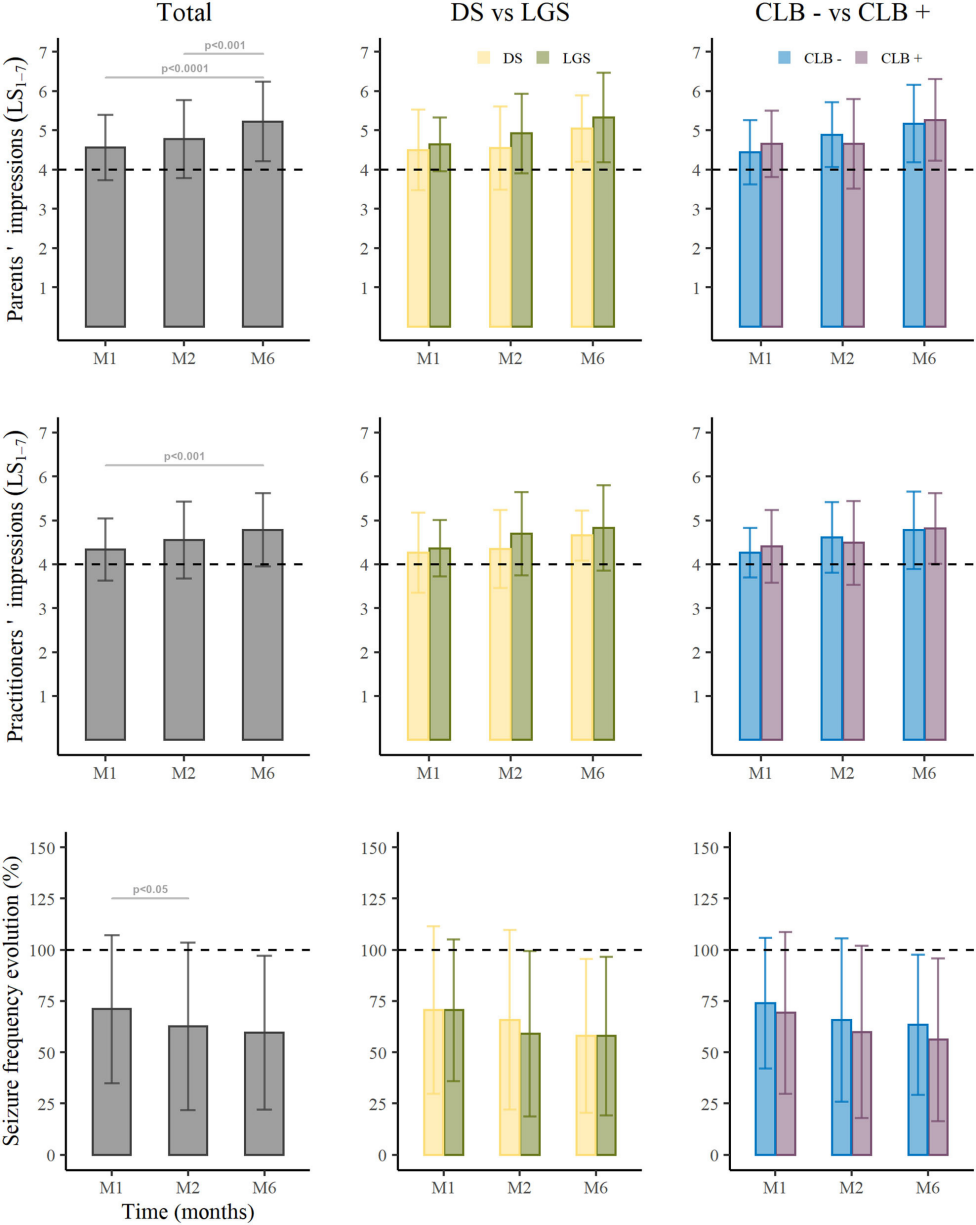
Cannabidiol efficacy in the total cohort, in the two subgroups of Dravet Syndrome and Lennox-Gastaut, and in patients CLB+ vs. CLB−. DS, Dravet Syndrome; LGS, Lennox-Gastaut syndrome; CLB− patients without clobazam, CLB+ patients in therapy with clobazam, *P < 0.05, **P < 0.01, ***P < 0.001.

Total seizure frequency change from baseline was −28.6% ± 36.1% at M1, −37.4% ± 40.9% at M2, and −41% ± 37.5% at M6. At M6, 28 patients (37.8%) had a reduction in total monthly seizures ≥50%, 23 (31.1%) had a reduction ≥70%, and six (8.1%) had a reduction ≥90% (Figure 5, left panel). One patient (DS) was seizure free from M1 and during the entire treatment period; two other patients (1 DS and 1 LGS) were seizure free at M6.

For patients with DS (*n* = 22), the reduction in monthly seizures at M6 was −43.9% ± 37.2% with 34.8% (*n* = 8) showing ≥50% reduction, 26.1% (*n* = 6) ≥70%, and 8.7% (*n* = 2) ≥90% (Figure 5, middle panel). For patients with LGS (*n* = 41), the reduction in monthly seizures at M6 was −42.1% ± 38.7% with 43.9% (*n* = 18) showing ≥50% reduction, 36.6% (*n* = 15) ≥70%, and 9.8% (*n* = 4) ≥90%. The seizure reduction responder rates and the reduction in monthly seizures did not differ significantly between these two subpopulations.

For CLB+ patients (*n* = 43), the reduction in monthly seizures at M6 was −42.7% ± 37.2% (Figure 5, right panel) with 43.2% (*n* = 19) showing a ≥50% reduction, 36.4% (*n* = 16) ≥70%, and 11.4% (*n* = 5) ≥90%. For CLB− patients (*n* = 30), the reduction in monthly seizures at M6 was −38.5% ± 38.5% with 31% (*n* = 9) showing a ≥50% reduction, 24.1% (*n* = 7) ≥70%, and 3.4% (*n* = 1) ≥90%. There was no significant difference in seizure reduction responder rates or percentage change in monthly seizures in these two subpopulations.

In multiple logistic regression analysis, none of the factors tested was statistically associated with the response rate (reduction ≥50% of all seizures). Moreover, there was no statistically significant difference between percentage reduction from baseline in seizure frequency and AE (OR 1.01, CI_95%_: 0.99–1.02, *p* = ns). In the CGI-I scale, parents' satisfaction with CBD add-on therapy was rated on average 4.56 ± 0.83 at M1, 4.77 ± 0.98 at M2, and 5.2 ± 1.0 at M6 with satisfaction at M6 being significantly higher compared to M1 (*p* < 0.001) and M2 (*p* = 0.007). Practitioners scored, on average, 4.34 ± 0.72 at M1, 4.55 ± 0.87 at M2, and 4.79 ± 0.83 at M6 with satisfaction at M6 significantly higher compared to M1 (*p* = 0.002). Parents' scores were higher than those of practitioners (Wilcoxon test: *p* < 0.001). Interestingly, there was significant correlation between parents and practitioner scores, both showing an increase over time (Pearson's correlation: 0.673; *p* < 0.001). CGI-I scores of parents were correlated negatively with seizure frequency (Pearson's correlation: −0.586; *p* < 0.001). Parents' scores were also significantly higher in patients without AE (OR 0.6, CI 95% 0.37–0.87, *p* < 0.001). No statistical difference in the CGI-I scores of parents and practitioners was found in the comparisons of the different subpopulations (DS vs. LGS and CLB+ vs. CLB−).

Twenty-four patients (19.2%) had ≤4 seizures per month at baseline, from one per trimester to three per month, and were not included in the efficacy analysis. At M6, 18 remained on CBD, and six stopped treatment. Sixteen experienced a reduction in seizure frequency; eight had a reduction ≥50% and the remainder ≥25%.

### Changes in Concomitant AEDs

Forty-seven patients (37.6%) reduced or suspended comedications, and 12% (*n* = 15) increased or introduced new AEDs. Reduction concerned mainly CLB (*n* = 19/47 patients, 40.4%), VPA (*n* = 14, 29.8%), and TPM (*n* = 7, 14.9%) leading to comedication total withdrawal in 25.5% of them (*n* = 12/47), including CLB (*n* = 2), TPM (*n* = 2), and also neuroleptics (*n* = 2). These comedication reductions occurred in 19.3% (*n* = 23 out of 119 patients assessed at M1), 22.1% (*n* = 23/104 at M2), and 17.2% (*n* = 15/87 at M6) of patients at M1, M2, and M6, respectively. They were due to AE in 57% at M1 (*n* = 13/23, including eight for somnolence and two for fatigue), 73.9% at M2 (*n* = 17/23 including nine for somnolence and five for fatigue), and 40% at M6 (*n* = 6/15 including one for somnolence and one for fatigue). Increase in doses of AEDs used in comedications or the administration of a new AED were reported in four patients at M1 (3.4%), four at M2 (3.8%), and eight at M6 (9.2%) due to partial or lack of efficacy. CBD was maintained in some of these cases for a benefit beyond seizure decrease as on behavior on interaction.

## Discussion

This is the first “real life” study that tested a slower titration of add-on CBD in a pediatric population with drug-resistant epilepsies. The profile of our population was close to those described in RCT and OL in terms of epileptic syndrome, sex ratio, number of AEDs per patient, age, and main treatments associated with CBD (Supplementary Table 1) (6–9, 11, 14–16). We showed lower reported AE and lower withdrawals because of AE with a similar efficacy compared to previous studies. Our results emphasized the lack of significant difference in efficacy or in AE rates between patients with DS or LGS and those receiving concomitant CLB (CLB+) or not (CLB−).

We demonstrated an improved safety profile compared to previous reports [48.8% vs. 74–93.4% (6, 7); for details see Supplementary Table 2]. Withdrawal due to AE was low in this study (1.6%) compared to 3–14% in previous studies (10, 14, 15). Patients reported less frequent AE, mainly decreased appetite [9.6% vs. 12.4%−28% (6, 16)], vomiting [0% vs. 3.7%–17.8% (12, 14)], and diarrhea [4.8% vs. 12.7%−34% (9, 11)]. A similar decrease in anorexia was reported in a slower titration of zonisamide, reducing the incidence of anorexia from 24.4 to 14.4% with a prolonged titration from 4 to 8 weeks (19). Somnolence and fatigue were within the same range as previously reported. Behavior disorders occurred in our study in 12.8% of patients, close to the higher range reported previously. Behavioral disorders were reported only in four studies with a rate between 3.5 and 11.9% (7, 8, 12, 14). Liver function enzymes were increased in 9.6% without any consequent withdrawal, putting this AE at the lower range of previous studies (7–23%) (10, 14). It occurred only in patients treated with both VPA and CLB.

Moreover, only a small proportion of patients had to stop CBD due to AE (1.6%). Our withdrawal rate (20.8%) was within the range of previous studies [7–28.4% (11, 14)]. Withdrawals were primarily due to CBD inefficacy and rarely to AEs (1.6%). Two factors could have contributed to these results. On one hand is a decrease in AE over time due to better patient tolerance due to slow titration of CBD. We aimed to reach a target dose of 10 mg/kg/day in at least 4 weeks compared to the dose of 20–25 mg/kg/day in 2–4 weeks used in the RCTs (Supplementary Table 2). Indeed, spontaneous improvement of AEs were observed during the treatment period in 33.3% at M2 and 52.6% at M6 without any change in comedications or CBD doses. The use of slower drug titration has been reported to limit AEs of many treatments, such as tramadol (20) and paroxetine (21) and also antiepileptic drugs, such as TPM (22), LMT (23), ZSD (19), and gabapentin (24). On the other hand, the slower titration of CBD allowed the modification of AED doses based on good clinical practice rules. Indeed, CBD titration, especially higher than 10 mg/kg/d, were decided by physicians according to patients' tolerance and efficacy. Reduction or suspension of comedication and CBD dose decrease yielded a disappearance of AEs in most cases and allowed the necessary time to test CBD efficacy. These data confirmed the importance of physician expertise in the management of AEDs and the benefit of applying the same rules for CBD use. We showed that physicians should not be too quick to stop CBD in the case of a mild AE because adapting treatments could avoid treatment discontinuation and give enough time to test efficacy. The risk–benefit ratio of CBD using the balance between efficacy and AEs, including liver function tests, should possibly be assessed frequently during titration to allow dose adjustments of CBD and associated comedications. This can control many AEs, limiting unnecessary withdrawal and allowing the evaluation of CBD efficacy and its potential benefit for the patient.

Efficacy was similar to that previously reported in OLE and RCTs (6, 9–12, 14–16) (Supplementary Table 3) and was similar for patients with DS and LGS. Interestingly, our results show no significant difference in efficacy for patients with CLB (CLB+) and without CLB (CLB−). This result could question the European Medicines Agency (EMA) (3) approval restricting CBD use to patients receiving CLB as “adjunctive therapy of seizures associated with LGS and DS, in conjunction with clobazam (CLB).” Given the number of AEDs being correlated with the presence of AEs, the addition of CLB to introduce CBD would probably increase the risk of AEs during the CBD titration. On the contrary, knowing the metabolic interaction and efficacy synergy between these two AEDs might help to privilege the use of CLB in association with CBD in the case of partial efficacy of the latter. We report a reduction in seizure frequency of 41% after 6 months of CBD with a responder rate of 38% (reduction ≥ 50%), similar to previous OL studies and RCTs (6, 8–12, 14, 16). Efficacy was similar in patients with DS [40–49.3% (8, 11)], LGS [59% (12)], and other etiologies, including TSC and other genetic epilepsies [37%−49% (14, 16)]. Caregivers' satisfaction with CBD was good and correlated to seizure reduction and also to the low levels of AEs.

Real-life studies are designed to analyze medication use under real-life conditions in clinical practice, following validation of the safety and efficacy of medicines in RCTs (25). They can extend the knowledge of a new drug and should be interpreted as complementary to RCTs (25). In our study, this type of design allowed us to evaluate the management of AE in clinical practice. It emphasized the need for comedication dose reduction to decrease AEs without a significant loss of efficacy in this cohort compared to RCTs. An unexpected result, probably due to the open-label design, was the difference of CBD doses between DS and LGS showing higher doses in DS. We might expect that higher doses would be needed more in LGS and not DS as CBD can increase the levels of STP, TPM, and CLB, the three drugs that are significantly more used in DS (26, 27). This might be explained by the difference of age between these two populations (patients with DS were younger than those with LGS). It cannot be excluded that higher doses of CBD may be required in younger children as per metabolism differences (28, 29). There are no comparisons of pharmacokinetic of CBD between children and adults to date due to limited data (4, 30).

Some limitations should be highlighted. The adaptation of the doses of comedications were based on clinical expertise and was not protocolized. Some centers decreased comedications faster than others. However, the reason for decreasing was often AEs. Another limitation could be the low number of patients (*n* = 125), but all French centers did not have the possibility to collect these cases in a voluntary initiative without any financial compensation. We believe that the prospective and multicentric aspects can strengthen the validity of these results because the design of this open-label study did not allow us to have a control group with a faster titration vs. our slow titration protocol. AEs were not quantified using quantitative scales but were reported as in clinical practice by parents and physicians. Finally, the comedication dose modifications were based on the physicians' expertise deciding which comedication to decrease or withdraw. We did not evaluate on a scale the severity of a given AE, but we might suppose that, for the most severe ones, actions such as decreasing comedications were undertaken. Finally, this study does not report the long-time outcome both for AE and efficacy. However, the follow-up was up to 27 weeks, longer than RCTs (up to 14 weeks) although shorter than the extension studies (38 to 48 weeks).

## Conclusion

Our study shows that a slower titration, whereby the target dose of 10 mg/kg/day is reached within at least 1 month, provides a better safety profile compared to previous RCT, OL, OLE, and EAP while providing similar efficacy. This could be due to the improved tolerance of the CBD by its slower introduction and also to the experience of practitioners who, through close clinical and biological monitoring of the patient, have adapted comedications, in particular CLB and VPA but also the dose of CBD. Efficacy was not significantly different between DS and LGS and between patients with and without CLB although, in Europe, CBD should be used in association with CLB. These data should be considered by physicians when initiating and using CBD therapy in drug-resistant epilepsies. A longer follow-up of this cohort will help to identify long-term retaining rates and AE.

## Data Availability Statement

The raw data supporting the conclusions of this article will be made available by the authors, without undue reservation.

## Ethics Statement

The studies involving human participants were reviewed and approved by Necker-enfants malades ethical committee. Written informed consent to participate in this study was provided by the participants' legal guardian/next of kin.

## Author Contributions

GD'O, MK, CH-L, BD, VS, SN, DV, J-MP, AL, CC, AS-M, TT, NC, MM, NV, and RN conceptualized and designed the study, coordinated, and supervised data collection. MK made the statistical analysis and designed the figures. All authors have critically revised the manuscript and approved the final one as submitted.

## Conflict of Interest

MM and NV moderated a symposium and a round table for GW pharma, for which he received an honorarium and reimbursement of transportation costs. The remaining authors declare that the research was conducted in the absence of any commercial or financial relationships that could be construed as a potential conflict of interest.
