# Supplementary material for: Slow Titration of Cannabidiol Add-On in Drug-Resistant Epilepsies Can Improve Safety With Maintained Efficacy in an Open-Label Study
Source: Front Neurol. 2020 Aug 12;11:829. doi: 10.3389/fneur.2020.00829 (PMC7434926; doi:10.3389/fneur.2020.00829)
Supplement: Supplementary file 1 [file Data_Sheet_1.docx]

Supplementary Material

**Supplementary Table 1: Comparison of baseline characteristics in previously published Cannabidiol open-label and randomized controlled trials.**

|  | ***Drug-resistant epilepsy*** | | | | ***Dravet syndrome*** | | | | | ***Lennox-Gastaut syndrome*** | | | |
| --- | --- | --- | --- | --- | --- | --- | --- | --- | --- | --- | --- | --- | --- |
|  | ***Devinsky***  ***2016***  (1) | ***Szaflarski***  ***2018***  (2) | ***Laux***  ***2019***  (3) | ***Our study*** | ***Devinsky***  ***2017***  (4) | ***Devinsky***  ***2018***  (5) | ***Devinsky***  ***2019***  (6) | ***Miller***  ***2020***  (7) | ***Our study*** | ***Devinsky***  ***2018***  (8) | ***Thiele***  ***2018***  (9) | ***Thiele***  ***2019***  (10) | ***Our study*** |
| Number of patients  (Safety/efficacy) | 162 / 137 | 607/580 | 152/147 | 125 | 61 | ***34*** | 264 | 133 | 48 | 73, 76 | 86 | 366 | 62 |
| Age (years) | 10.5 (0.9-26.2) | 13.2 (0.4‐62.1) | 12 (1.7-51) | 9 [6-14] | 9.1 [2.5–18.0] | 7.6±1.8 | 9.3 (2.5-19.3) | 9.2 (2.3-17.7)  9.3 (2.2-18.9) | 7 [5-10.3] | 15.4 ± 9.5  16 ± 10.8 | 14.2 (2.7–39.0) | 13.7 (3.0‐48.3) | 13 [8.25-15.8] |
| Gender (male) | 80 (49%) | 313 (52%) | 93 (61%) | 59 (47.2%) | 35 (57%) | *16 (47%)* | 133 (50%) | 27 (41%)  36 (54%) | 18 (37.5%) | 40 (55%),  45 (59%) | 45 (52%) | 198 (54%) | 35 (56.4%) |
| **Dravet** | 33 (20%) | 58 (10%) | 58 (39%) | 48 (38.4%) | 61 (100%) | 34 (100%) | 264 (100%) | 66 (100%)  67 (100%) | 48 (100%) | - | - | - | - |
| **Lennox-Gastaut** | 31 (19%) | 94 (15%) | 94 (61%) | 62 (49.6%) | - | - | - | - | - | 149 (100%) | 86 (100%) | 366 (100%) | 62 (100%) |
| Number of AEDs | 3 (0-7) | 3 (0‐10) | 3 (0-10) | 3 [3-4] | 3.0±1.0 | 2.6 ± 0.9 | 3 (1-6) | 3 (1-5)  3 (1-4) | 3 [3-4] | 3 [1–5],  3 [0–5] | 3 [1–5] | 3 (0-9) | 3 [3-4] |
| CLB | 85 (52%) | 319 (53%) | 99 (65.1%) | 76 (60.8%) | 40 (66%) | 23 (68%) | 180 (68%) | 46 (68%)  40 (60%) | 37 (77.1%) | 37 (51%),  36 (47%) | 41 (48%) | 188 (51%) | 33 (53.2%) |
| VPA | 48 (30%) | 183 (30%) | 64 (42.1%) | 81 (64.8%) | 37 (61%) | 22 (65%) | 168 (64%) | 44 (67%)  47 (70%) | 81 (64.8%) | 27 (37%),  28 (37%) | 36 (42%) | 136 (37%) | 40 (64.5%) |
| STP | - | 16 (3%) | - | 38 (30.4%) | 30 (49%) | 7 (21%) | 101 (38%) | 25 (38%)  22 (33%) | 38 (79.2%) | - | - | - | 0 (0%) |
| VNS | 17 (10%) | 18 (3%) | - | 16 (13%) | 6 (10%) | 2 (6%) | - | - | 0 (0%) | 15 (21%),  17 (22%) | 26 (30%) | - | 14 (23%) |
| KD | 13 (8%) | 18 (3%) | - | 5 (4%) | 6 (10%) | 6 (18%) | - | - | 2 (4.1%) | 6 (8%),  6 (8%) | 4 (5%) | - | 1 (2%) |
| Monthly frequency of TOTAL seizures | 60.5  [19.6-151] | 72*  [22-196] | 63 [23-140] | 30.5  [5-122] | 24.0 | - | 32.4 | 35 (10-104)  26 (10-194) | 5.5  [3.9-13.5] | 165.0 [81.3-359],  174.3 [82.7-392.4] | 144.6  [72.0-385.7] | 167.6 | 91.5  [38.3-236.4] |

Data are n (%), mean ± standard deviation, median (range) or median [^25th-75th^ percentile] and based on analysis group.

*Based on efficacy group.

**Supplementary Table 2. Comparison of Cannabidiol safety in previously published open-label and randomized controlled trials.**

|  | ***Drug-resistant epilepsy*** | | | | ***Dravet syndrome*** | | | | | ***Lennox-Gastaut syndrome*** | | | |
| --- | --- | --- | --- | --- | --- | --- | --- | --- | --- | --- | --- | --- | --- |
|  | ***Devinsky***  ***2016***  (1) | ***Szaflarski***  ***2018***  (2) | ***Laux***  ***2019***  (3) | ***Our study*** | ***Devinsky***  ***2017***  (4) | ***Devinsky***  ***2018***  (5) | ***Devinsky***  ***2019***  (6) | ***Miller***  ***2020***  (7) | ***Our study*** | ***Devinsky***  ***2018***  (8) | ***Thiele***  ***2018***  (9) | ***Thiele***  ***2019***  (10) | ***Our study*** |
| Study | OL | EAP | EAP | OL | RCT | RCT | OLE | RCT | OL | RCT | RCT | OLE | OL |
| Period | *01/2014 - 01/2015* | *01/2014 - 12/2016* | *01/2014-12/2016* | *03/2019-09/2019* | *-* | *10/2014-03/2015* | *11/2016* | *04/2015-11/2017* | *03/2019-09/2019* | *06/2015 - 12/2015* | *04/2015 - 10/2015* | *11/2016* | *03/2019-09/2019* |
| Number of patients | 162 | 607 | 152 | 125 | 61 | 10, 8, 9 | 264 | 66, 67 | 48 | 67, 82 | 86 | 366 | 62 |
| Age of patients | 1-30 y | - | 1.7-51 | 2-20 y | 2-18 y | 4-10 y | 2-18 y | 2-18 y | 2-17 y | 2-55 y | 2-55 y | 2-55 y | 2-20 y |
| CBD titration | 2-5 mg/kg/w | 2-10 mg/kg/d | 2-10 mg/kg/d | 2.5 mg/kg/w | 10 mg/kg/w | - | 10 mg/kg/w | 1.1 mg/kg/d  1.6 mg/kg/d | 2.5 mg/kg/w | 2.5-5 mg/kg/d | 10 mg/kg/w | 10 mg/kg/w | 2.5 mg/kg/w |
| CBD max dose (mg/kg/d) | 25-50 | 25-50 | 25-50 | 25 | 20 | 20 | 20-30 | 10, 20 | 20 | 10, 20 | 20 | 20-30 | 20 |
| CBD mean/median (mg/kg/d) | 22.9 ± 9.1 | 25 | 25 | 13.5 ± 4.2 | 20 | 5, 10, 20 | 21.2 ± 5.2 | 10, 20 | 14 ± 3.6 | 10, 20 | 20 | 22.8 ± 5 | 13.1 ± 4.5 |
| Study lenght | 12 w | 48 w [23-95] | 80.1 [20.7-107.7] | 27 w | 14 w | 3 w | 274 d (1-512) | 14 w | 27 w | 14 w | 14 w | 38 w (3-430 d) | 27 w |
| **Withdrawal** | 11 (6.8%) | 146 (24%) | 42 (27.6%) | 26 (20.8%) | 9 (14.7%) | 0, 1, 1 (7.4%) | 75 (28.4%) | 9 (6.7%) | 11 (23 %) | 2, 9 (7.4%) | 14 (16.3%) | 67 (18.3%) | 10 (16.1%) |
| AE | 5 (3%) | 31 (5.1%) | 5 (3.3%) | 2 (1.6%) | 8 (13.1%) | 2 (7.4%) | 19 (7.2%) | 5 (3.7%) | 1 ( 2%) | 1, 6 (4.7%) | 12 (14%) | 35 (9.6%) | 0 (0%) |
| Hepatoxicity | 1 (<1%) | - | ? | 0 (0%) | - | - | - |  | 0 (0%) | - | 2 (2.3%) | - | 0 (0%) |
| AST, AST | - | - | ? | 0 (0%) | 3 (4.9%) | 1 (3.7%) | 10 (3.8%) |  | 0 (0%) | 1, 4 (3.3%) | 6 (7%) | 5 (1.4%) | 0 (0%) |
| **AE, n (%)** | 128 (79%) | 536 (88.3%) | 138 (91.8%) | 61 (48.8%) | 57 (93.4%) | 8, 5, 7 (74%) | 246 (93.2%) |  | 30 (62.5 %) | 56, 77 (89.3%) | 74 (86%) | 337 (92.1%) | 26 (41.9%) |
| Somnolence | 41 (25%) | 136 (22.4%) | 45 (30%) | 26 (20.8%) | 22 (36%) | 2, 3, 0 (18.5%) | 65 (24.6%) | 16 (25%)  16 (23%) | 11 (23%) | 14, 25 (26.2%) | 13 (15%) | 86 (23.5%) | 13 (21%) |
| Decr. appetit | 31 (19%) | 75 (12.4%) | 24 (16%) | 12 (9.6%) | 17 (28%) | 0, 1, 4 (18.5%) | 67 (25.4%) | 11 (17%)  20 (29%) | 8 (16.7%) | 11, 21 (21.5%) | 11 (13%) | 65 (17.8%) | 3 (4.8%) |
| Diarrhea | 31 (19%) | 177 (29.2%) | 36 (24%) | 6 (4.8%) | 19 (31%) | - | 91 (34.5%) | 11 (17%)  18 (26%) | 5 (10.4%) | 7, 12 (12.7%) | 16 (19%) | 98 (26.8%) | 1 (1.6%) |
| Fatigue | 21 (13%) | 65 (10.7%) | 24 (16%) | 20 (16%) | 12 (20%) | 0, 0, 1 (3.7%) | 27 (10.2%) | 5 (8%)  15 (22%) | 9 (18.8%) | - | 9 (10.5%) | - | 8 (12.9%) |
| Behaviour disorder | 6 (3.7%) | - | - | 16 (12.8%) | - | 3, 0, 0 (11.1%) | - | 3 (3.8%)  5 (11.9%) | 6 (12.5%) | - | 3 (3.5%) | - | 9 (14.5%) |
| Vomiting | 6 (3.7%) | 69 (11.4%) | 20 (13%) | 0 (0%) | 9 (15%) | 1, 1, 1 (11.1%) | 37 (14%) | 4 (6%)  11 (16%) | 0 (0%) | 4, 10 (9.4%) | 9 (10.5%) | 65 (17.8%) | 0 (0%) |
| Convulsion | 18 (11%) | 102 (17%) | 36 (24%) | 1 (0.8%) | 7 (11%) | 0, 1 (3.7%), 0 | 40 (15.2%) | 5 (8%)  7 (10%) | 1 (2%) | - | 5 (5.8%) | 78 (21.3%) | 0 (0%) |
| Liver function tests | 11 (7%) | 61 (10%) | 22 (15%) | 11 (9.6%) | 12 (19.7%) | 1, 1, 4 (22.2%) | 22/128 (17.2%) | 3 (5%)  9 (13 %) | 7 (14.6%) | 3, 11 (9.4%) | 20 (23.2%) | 37 (10%) | 4 (6.5%) |

Data are n (%), mean ± standard deviation or median (range), [^25th-75th^ percentile].

Legend: CBD Cannabidiol, OL Open label, EAP Expanded Access Program, RCT Randomized controlled trial, OLE Open label extension, w week, d day, y years, CBD max maximum dose, AE adverse events, Decr. appetit decreased appetit.

**Supplementary Table 3. Comparison of Cannabidiol efficacy in previously published open-label and randomized controlled trials.**

One study was excluded because it lacks efficacy data^4^.

|  | ***Drug-resistant epilepsy*** | | | | ***Dravet syndrome*** | | | | ***Lennox-Gastaut syndrome*** | | | |
| --- | --- | --- | --- | --- | --- | --- | --- | --- | --- | --- | --- | --- |
|  | ***Devinsky***  ***2016***  (1) | ***Szaflarski***  ***2018***  (2) | ***Laux***  ***2019***  (3) | ***Our study*** | ***Devinsky***  ***2017***  (4) | ***Devinsky***  ***2019***  (6) | ***Miller***  ***2020***  (7) | ***Our study*** | ***Devinsky***  ***2018***  (8) | ***Thiele***  ***2018***  (9) | ***Thiele***  ***2019***  (10) | ***Our study*** |
| Number of patients | 137 | 580 | 147 | 125 | 61 | 104 | 67  67 | 48 | 73  76 | 86 | 366 | 62 |
| CBD dose  mean/median | 22.7 mg/kg/d  (± 8.5) | 25 mg/kg/d | 25 mg/kg/d | 13.5 ± 4.2 mg/kg/d | 20 mg/kg/d | 21 mg/kg/d | 10 mg/kg/d,  20 mg/kg/d | 14 ± 3.6 mg/kg/d | 10 mg/kg/d,  20 mg/kg/d | 20 mg/kg/d | 22.8 mg/kg/d  (± 8.5) | 13.1 ± 4.5 mg/kg/d |
| CONVULSIVE  seizures | - | -51% (at 12 w) | - |  | -38.9 %  [-69.5 to -4.8] | -44% (37-48w)  [-83 to -5] | -48.7% (14w)  -45.7% (14w) |  | - | - | - |  |
| DROP  seizures | - | - | - |  | - | - | - |  | -37.2%,  -41.9% | -43.9%  [-69.6 to -1.9] | -60% (37-48 w)  [-88 to -23] |  |
| TOTAL seizures  (monthly frequency) | -34.6%  [-66.7 to -9.8] | -48% (at 12 w) | -41 -46%  (12w to 96 w) | -41± 37.5% at 27 w | -28.6%  [-70 to -4] | -39.5% (37-48w)  [-86 to +7] | - 56.4% (14 w)  - 47.3% (14 w) | -43.9 ± 37.2%. at 27 w | -36.4%,  -38.4% | -41.2%  [-62.9 to -13] | -57% (37-48 w)  [-82 to -22] | -42.1± 38.7% at 27 w |
| TOTAL seizures  (≥50% reduction) | 51 (37%) | 211/431 (49%)  (at 12 w) | Between 43 and 50% | 28 (37.8%) at 27 w | - | 42 (40%)  (37-48w) | 29 (43.9%)  33 (49.3%) | 8 (34.8%) at 27 w | - | - | 123/209 (59%)  (37-48 w) | 18 (43.9%) at 27 w |

Data are n (%), mean ± standard deviation or median [25th to 75th percentile].

Legend: CBD Cannabidiol, CGI-I Clinical Global Impression-Improvement.

**Bibliography:**

1. Devinsky O, Marsh E, Friedman D, Thiele E, Laux L, Sullivan J, Miller I, Flamini R, Wilfong A, Filloux F, et al. Cannabidiol in patients with treatment-resistant epilepsy: an open-label interventional trial. *Lancet Neurol* (2016) **15**:270–278. doi:10.1016/S1474-4422(15)00379-8

2. Szaflarski JP, Bebin EM, Comi AM, Patel AD, Joshi C, Checketts D, Beal JC, Laux LC, De Boer LM, Wong MH, et al. Long-term safety and treatment effects of cannabidiol in children and adults with treatment-resistant epilepsies: Expanded access program results. *Epilepsia* (2018) **59**:1540–1548. doi:10.1111/epi.14477

3. Laux LC, Bebin EM, Checketts D, Chez M, Flamini R, Marsh ED, Miller I, Nichol K, Park Y, Segal E, et al. Long-term safety and efficacy of cannabidiol in children and adults with treatmentresistant Lennox-Gastaut syndrome or Dravet syndrome: Expanded access program results. *Epilepsy Res* (2019) **154**:13–20. doi:10.1016/j.eplepsyres.2019.03.015

4. Devinsky O, Cross JH, Laux L, Marsh E, Miller I, Nabbout R, Scheffer IE, Thiele EA, Wright S. Trial of cannabidiol for drug-resistant seizures in the dravet syndrome. *N Engl J Med* (2017) **376**:2011–2020. doi:10.1056/NEJMoa1611618

5. Devinsky O, Patel AD, Thiele EA, Wong MH, Appleton R, Harden CL, Greenwood S, Morrison G, Sommerville K. Randomized, dose-ranging safety trial of cannabidiol in Dravet syndrome. *Neurology* (2018) **90**:e1204–e1211. doi:10.1212/WNL.0000000000005254

6. Devinsky O, Nabbout R, Miller I, Laux L, Zolnowska M, Wright S, Roberts C. Long-term cannabidiol treatment in patients with Dravet syndrome: An open-label extension trial. *Epilepsia* (2019) **60**:294–302. doi:10.1111/epi.14628

7. Miller I, Scheffer IE, Gunning B, Sanchez-Carpintero R, Gil-Nagel A, Perry MS, Saneto RP, Checketts D, Dunayevich E, Knappertz V. Dose-Ranging Effect of Adjunctive Oral Cannabidiol vs Placebo on Convulsive Seizure Frequency in Dravet Syndrome: A Randomized Clinical Trial. *JAMA Neurol* (2020) doi:10.1001/jamaneurol.2020.0073

8. Devinsky O, Patel AD, Cross JH, Villanueva V, Wirrell EC, Privitera M, Greenwood SM, Roberts C, Checketts D, VanLandingham KE, et al. Effect of cannabidiol on drop seizures in the lennox–gastaut syndrome. *N Engl J Med* (2018) **378**:1888–1897. doi:10.1056/NEJMoa1714631

9. Thiele EA, Marsh ED, French JA, Mazurkiewicz MB, Benbadis SR, Joshi C, Lyons PD, Taylor A, Roberts C, Sommerville K, et al. Cannabidiol in patients with seizures associated with Lennox-Gastaut syndrome (GWPCARE4): a randomised, double-blind, placebo-controlled phase 3 trial. *Lancet* (2018) **391**:1085–1096. doi:10.1016/S0140-6736(18)30136-3

10. Thiele E, Marsh E, Mazurkiewicz-Beldzinska M, Halford JJ, Gunning B, Devinsky O, Checketts D, Roberts C. Cannabidiol in patients with Lennox-Gastaut syndrome: Interim analysis of an open-label extension study. *Epilepsia* (2019) **60**:419–428. doi:10.1111/epi.14670
